# Supplementary material for: BaCoN (Balanced Correlation Network) improves prediction of gene buffering
Source: Mol Syst Biol. 2025 Apr 22;21(7):807–24. doi: 10.1038/s44320-025-00103-7 (PMC12222496; doi:10.1038/s44320-025-00103-7)
Supplement: Supplementary file 1 — Appendix [file 44320_2025_103_MOESM1_ESM.pdf]

Appendix for

BaCoN (Balanced Correlation Network) improves prediction of gene buffering

Thomas Rohde[1], Talip Yasir Demirtas[1], Sebastian Suesser[2], Angela Helen Shaw[1], Manuel Kaulich[2], Maximilian Billmann[1+]

Affiliation

[1] Institute of Human Genetics, University of Bonn, School of Medicine and University Hospital Bonn, Bonn, 53127, Germany
[2] Institute of Biochemistry II, Faculty of Medicine, Goethe University Frankfurt, Theodor-Stern-Kai 7, 60590, Frankfurt am Main, Germany

[+]correspondence to maximilian.billmann@gmail.com

Table of contents

Appendix Figure S1. Transformation of buffering prediction scores when utilizing different methods to normalize gene effect and expression data. 2

Appendix Figure S2. True positive metrics for buffering prediction evaluation and BaCoN correction factor selection. 3

Appendix Figure S3. Biases in uncorrected, PCC-based buffering prediction and metric quantifying likely false positive predictions. 4

Appendix Figure S4. Number of gene pairs in several sub-categories among the top predictions using different normalization methods. 5

Appendix Figure S5. False discovery rates for top 1000 predictions for the tested methods of predicting buffering. 6

Appendix Figure S6. Prediction characteristics for random subsets of cell lines. 7

Appendix Figure S7. Network visualization of the top 100 buffering predictions using 10 random samples from the 1019 DepMap cell lines. 8

Appendix Figure S8. BaCoN performance for predicting gene buffering using different omics data sets from the Cancer Dependency Map. 9

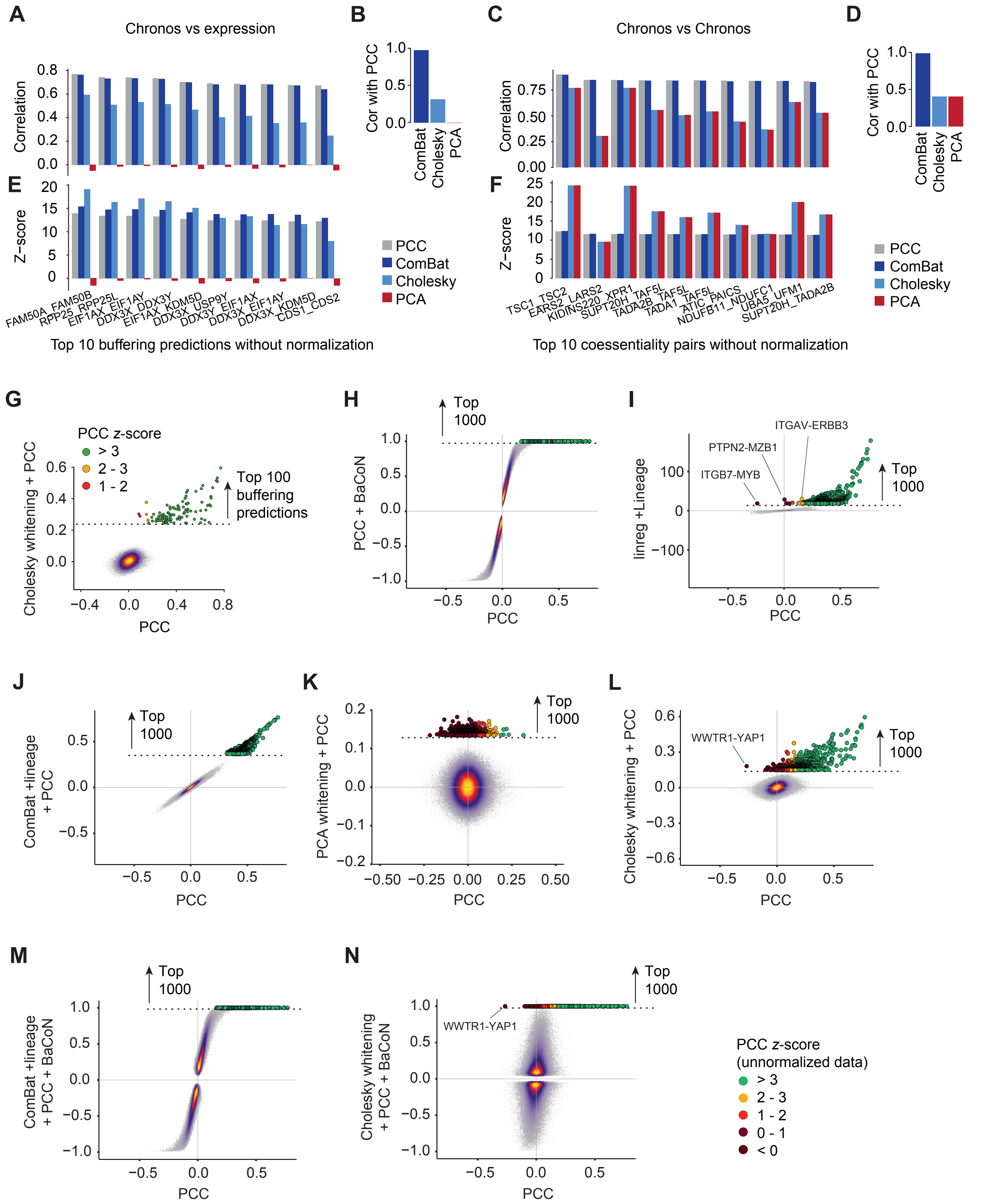

Appendix Figure S1. Transformation of buffering prediction scores when utilizing different methods to normalize gene effect and expression data. All methods have been performed on the approximately 147 million gene pairs. **A.** Changes in Chronos scores vs expression PCC for the 10 gene pairs with the strongest PCC before normalization. The PCCs for each pair before normalization and after the application of three a priori normalization methods shows loss of Chronos score vs expression covariance after PCA whitening. **B.** Global correlation between 147 million unnormalized (PCC) and normalized (ComBat, Cholesky, PCA) buffering predictions. **C.** The changes in co-essentiality PCCs (Chronos vs Chronos) for the 10 gene pairs with the strongest PCC before normalization. Shown are the PCC for each pair before normalization and after the application of three a priori normalization methods**. D.** Global correlation between 141 million unnormalized (PCC) and normalized (ComBat, Cholesky, PCA) co-essentiality scores**. E.** and **F.** Z-transformed scores from A. and B. G. Cholesky whitening plus PCC-based compared to PCC only. All scores are shown as heatscatter. The top 100 Cholesky whitening plus PCC predictions are shown larger and color-coded by z-score bin of the PCC only scores**. H. – N.** Selected normalization method-based predictions compared to PCC only. All scores are shown as heatscatter. The top 1000 corrected predictions are shown larger and color-coded by z-score bin of the PCC only scores. Selected strongly transformed gene pairs are labeled.

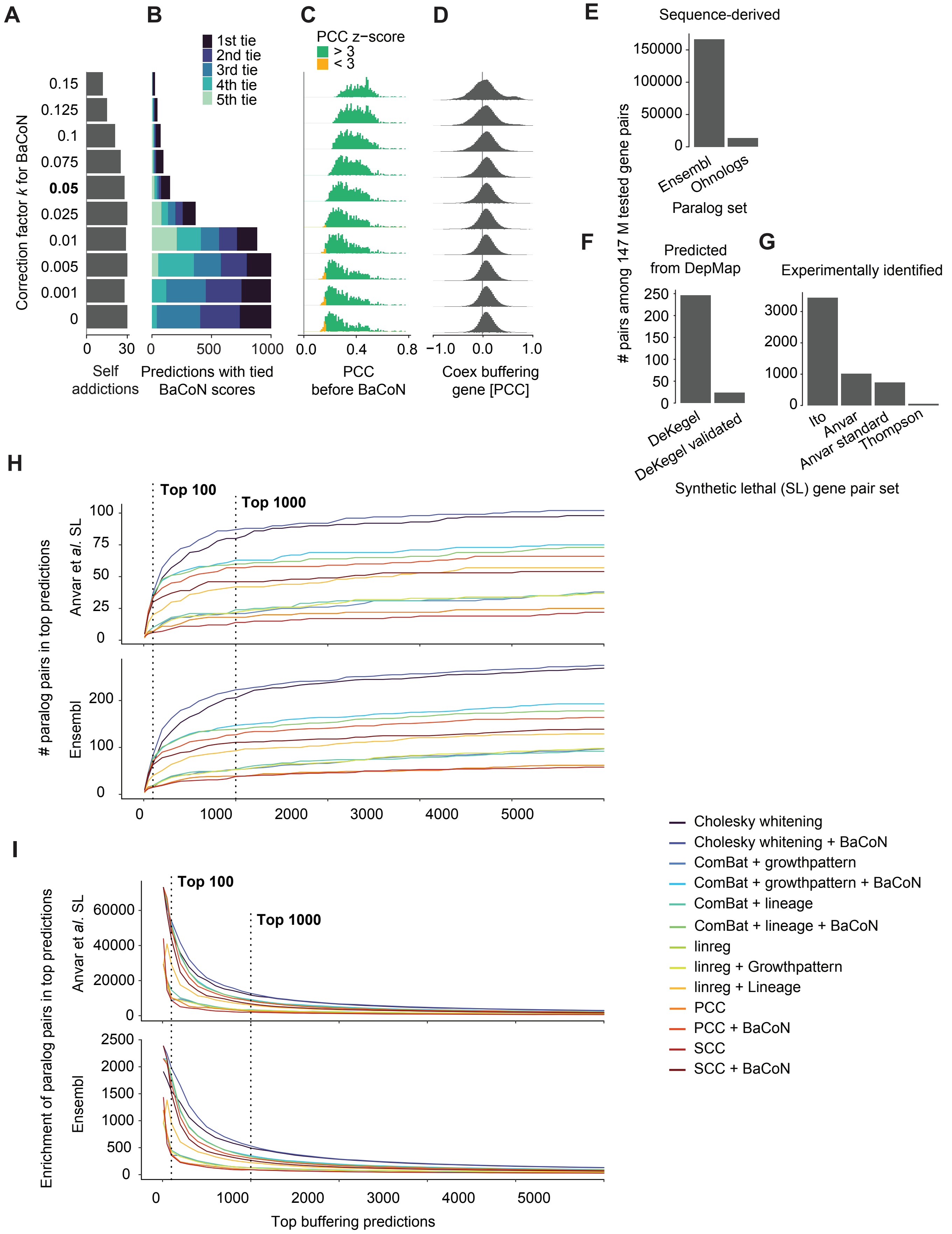

Appendix Figure S2. True positive metrics for buffering prediction evaluation and BaCoN correction factor selection. A. Number of self-addictions among the top 1000 predictions. **B.** Number of predictions with top 1 – 5 score ties among the top 1000 predictions**. C.** Data transformation strength of BaCoN indicated by the PCC prior to normalization of the top 1000 BaCoN predictions. **D.** Co-expression among all buffering genes. **E.** Number of Ensembl and Ohnolog paralog pairs in the universe. **F.** Number of predicted synthetic lethal (SL) gene pairs in the universe**. G.** Number of experimentally identified SL paralog pairs in different studies. **H.** Number of Ensembl paralog pairs and experimentally identified SL pairs among the up to 5000 top predictions using different normalization methods alone or in combination. The top 100 and 1000 predicted pairs are marked. **I.** Foldchange (number over all predicted) of Ensembl paralog pairs and experimentally identified SL pairs among the up to 5000 top predictions using different normalization methods alone or in combination. The top 100 and 1000 predicted pairs are marked.

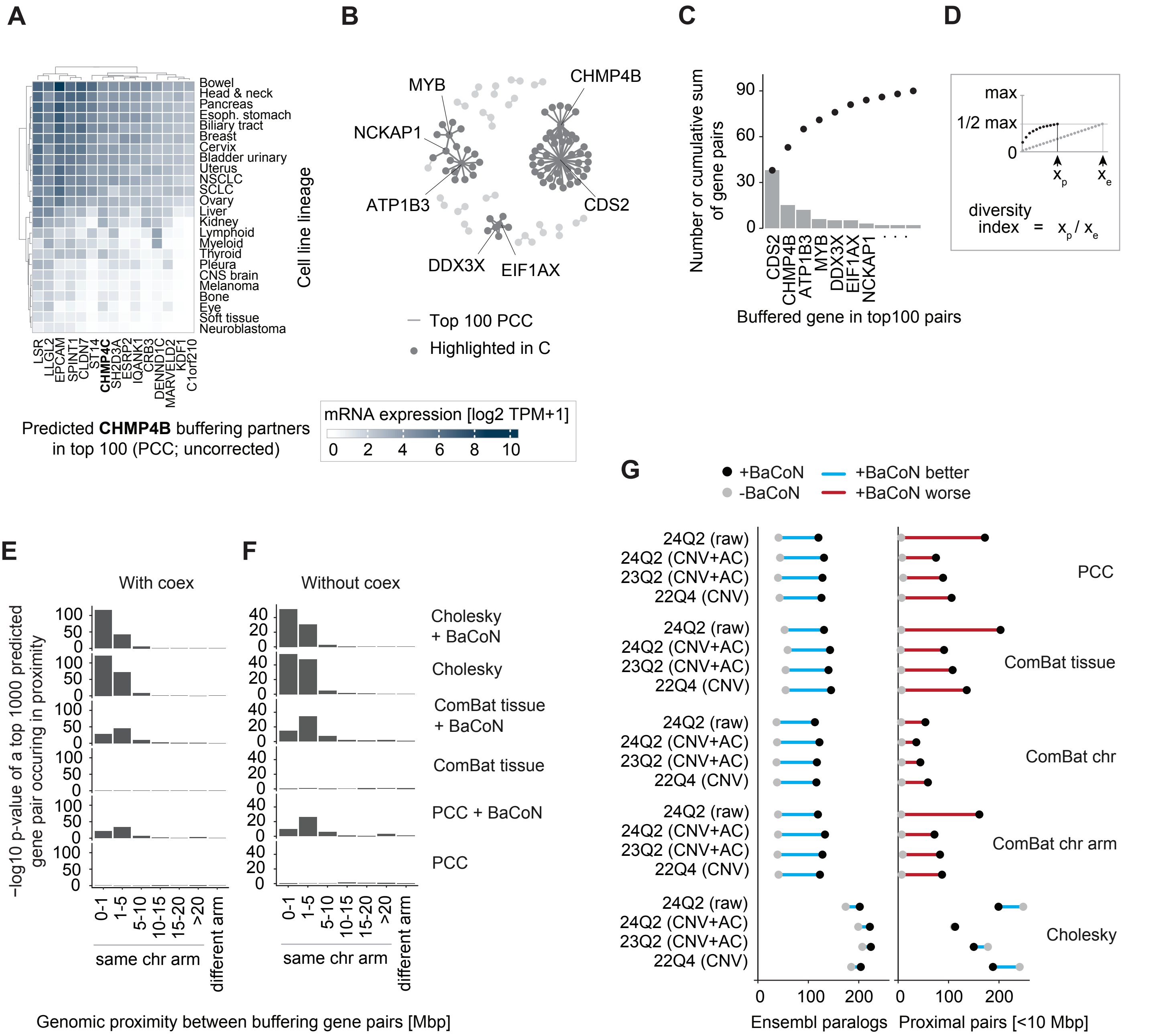

Appendix Figure S3. Biases in uncorrected, PCC-based buffering prediction and metric quantifying likely false positive predictions.
A. Mean expression in cell lines derived from the indicated lineages for all genes connected to CHMP4B among the top 100 buffering predictions shown in B**.** **B.** Network of the PCC-based top 100 buffering predictions. Genes that are predicted to be buffered by at least three genes are labeled. **C.** Number of top 100 pairs a gene is involved in (bars) and cumulative sum of pairs (dots). Genes that are predicted to be buffered by at least three genes are labeled. **D.** Illustration of the diversity index when defining xp (number of genes) at the half maximum number, where xe is the maximum expected number of e.g. 200 genes in top 100 pairs**.** **E.** and **F.** Enrichment of buffering predictions on the same chromosome, within different proximity windows, among the top 1000 buffering predictions considering all genes (E) or gene without co-expression false positives (F). The enrichments are computed using the negative logarithmic p-value of a hypergeometric test, comparing the density of proximal pairs among the predictions with the global density of pairs for each respective window of proximity**.** **G.** Proximity bias and paralog detection performance of normalization methods with and without BaCoN on Chronos score data with and without copy number variation (CNV) correction and chromosomal arm correction (AC).

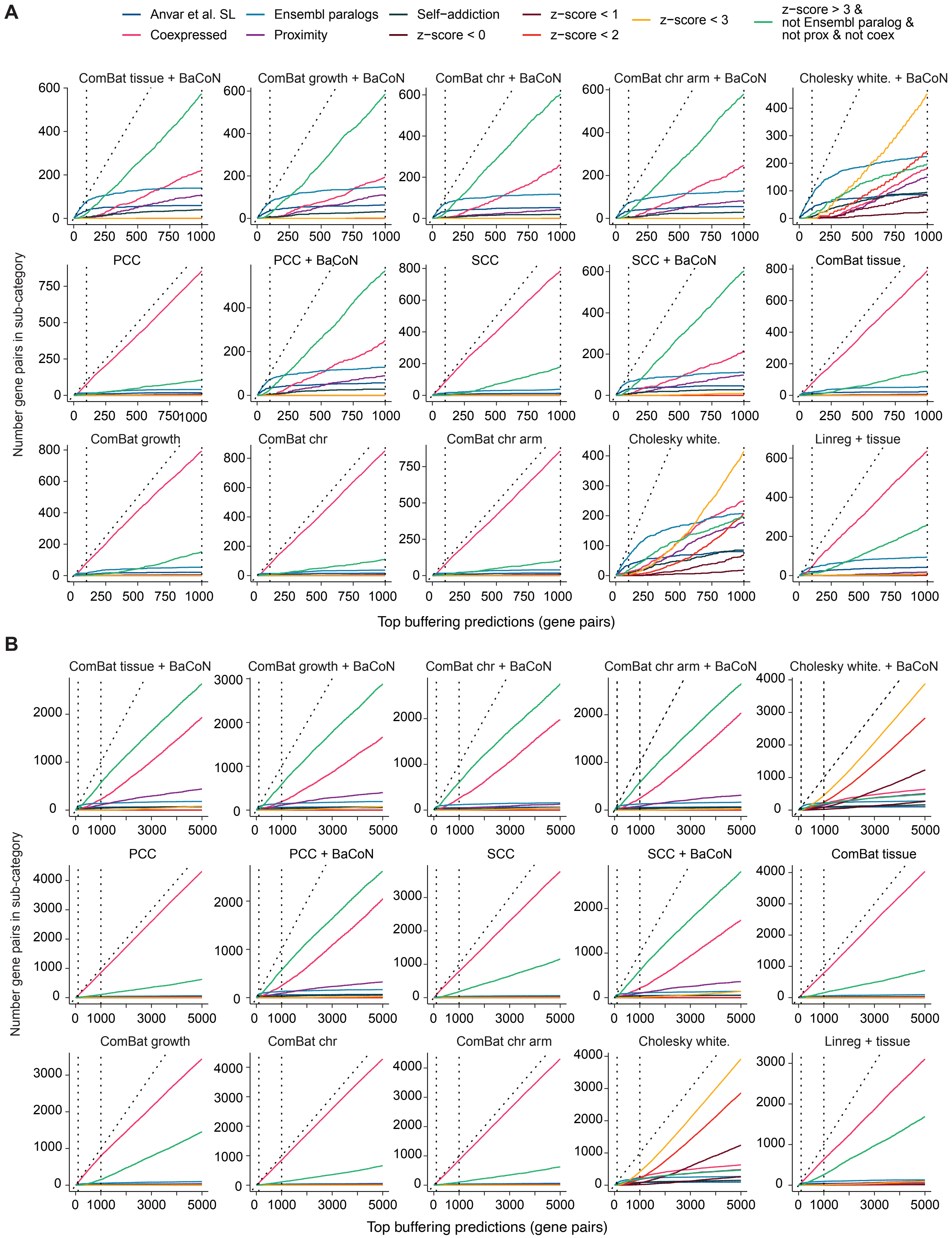

Appendix Figure S4. Number of gene pairs in several sub-categories among the top predictions using different normalization methods. Sub-categories are the number of Ensembl gene pairs, experimentally identified SL gene pairs, number of highly correlated pairs that are not Ensembl paralogs, number of self-addictions and the number of pairs with low correlation in the original data at different thresholds. **A.** Number of gene pairs of each sub-category among the up to top 1000 predictions. The dotted diagonal is the maximum value a sub-category can reach. The high-confidence (top 100) and standard (top 1000) prediction thresholds are marked**. B.** Number of gene pairs of each sub-category among the up to top 5000 predictions.

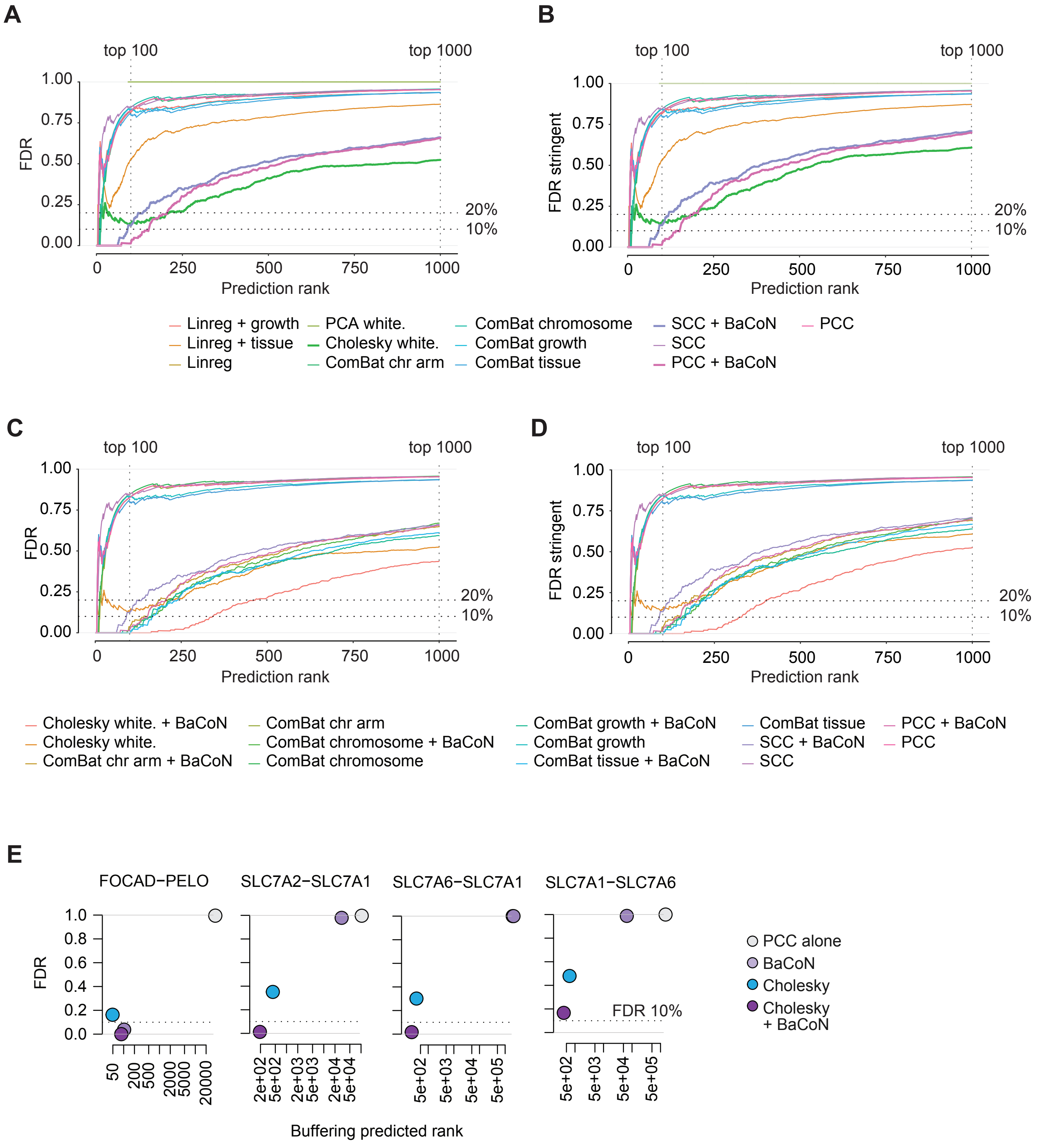

Appendix Figure S5. False discovery rates for top 1000 predictions for the tested methods of predicting buffering. A. and **C.** Performance of normalization methods with (A) and without (C) BaCoN using an empirical FDR which summarized five of our six performance metrics. This standard empirical FDR was used throughout this manuscript unless noted otherwise. The FDR considering Ensembl paralogs, Ohnologs as well as experimentally validated pairs and self-addictions as true positive (TP) predictions and proximal pairs and pairs driven by co-expression of the buffering gene with stronger connected buffering genes as false positive (FP) predictions. Panel A corresponds to Fig. 2H. **B.** and **D.** Performance of normalization methods with (B) and without (D) BaCoN using a stringent empirical FDR which summarized five of our six performance metrics. The stringent FDR was computed analogously, but without self-addictions as TP. FP sets were kept identical for both the stringent and the non-stringent FDR. Panel C corresponds to Fig. 2G**. E.** FDR and buffering prediction rank for gene pairs recently discovered in (Wolf et al, 2024; Borck et al, 2025).

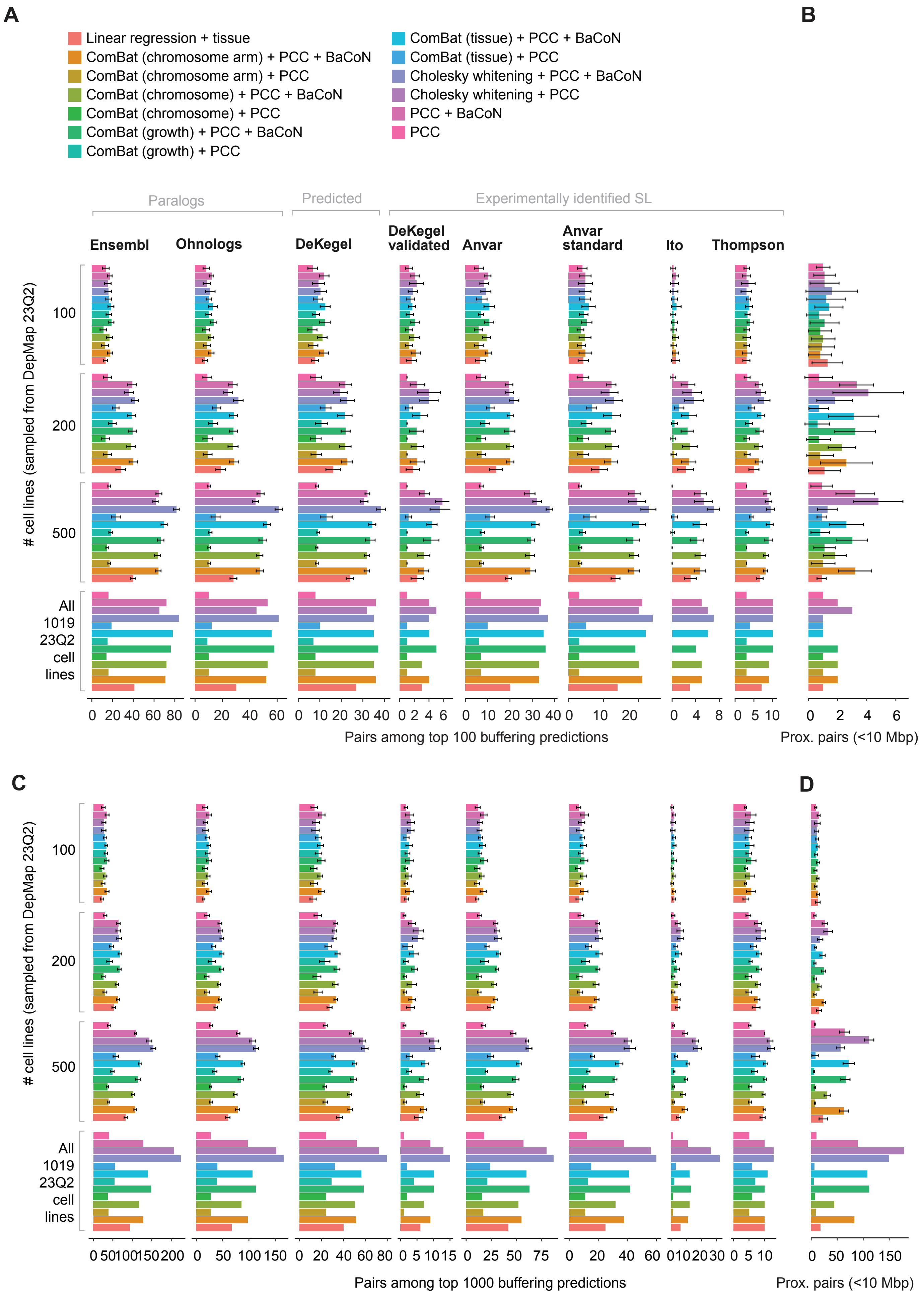

Appendix Figure S6. Prediction characteristics for random subsets of cell lines. This figure accompanies Figure 4A and shows the mean number of several categories of top 100 predictions of all tested methods. Each method was tested on ten independent pseudo-random cell line subsets of 100, 200 as well as 500 cell lines. **A.** and **C.** Number of Ensembl paralogs, Ohnologs as well as predicted and experimentally validated SL pairs among top 100 (A) and top 1000 (C) predictions. **B.** and **D.** Number of proximal pairs (less than 10 million base pairs apart) among top 100 (B) and top 1000 (D) predictions.

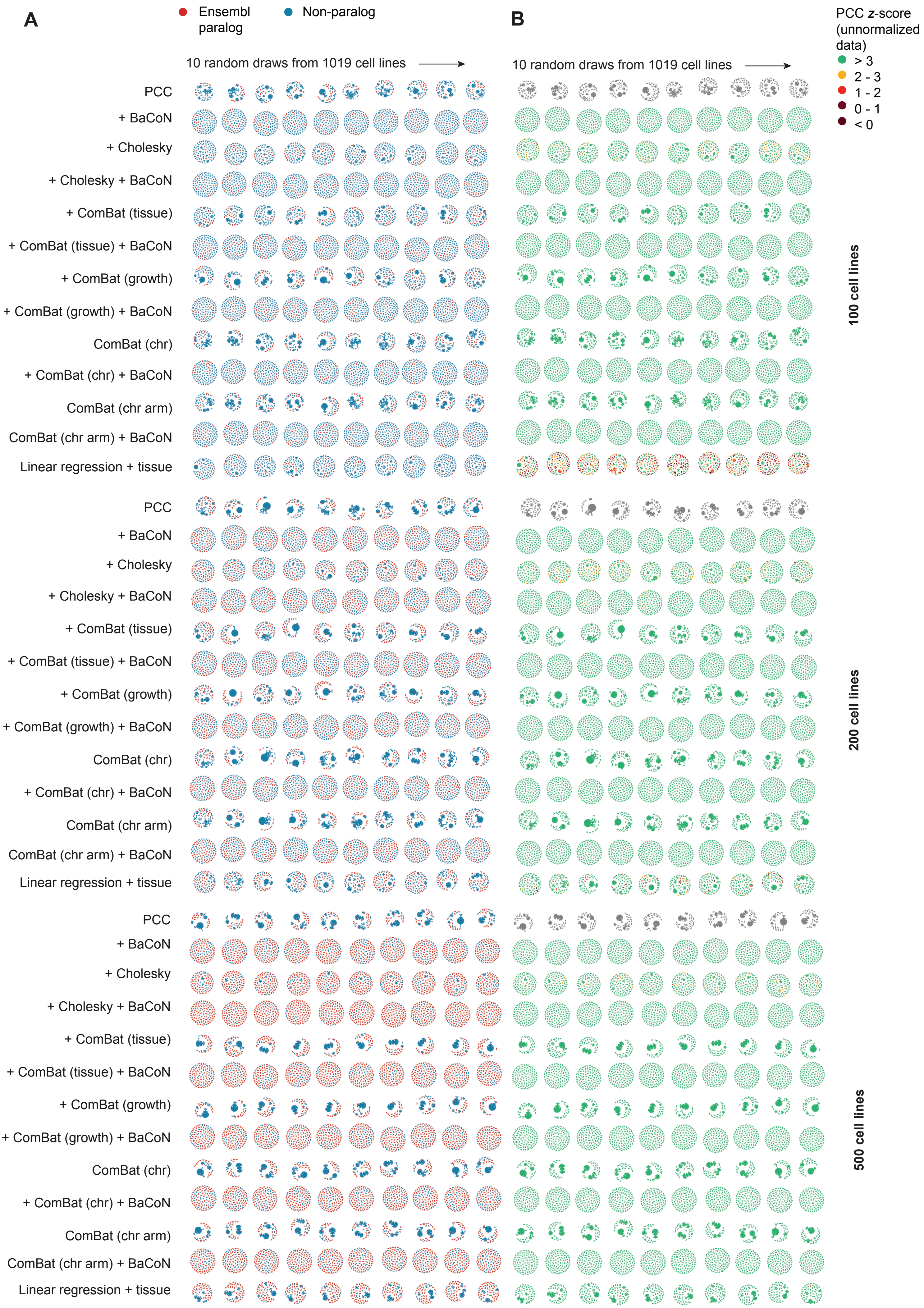

Appendix Figure S7. Network visualization of the top 100 buffering predictions using 10 random samples from the 1019 DepMap cell lines. This figure accompanies Figure 4C providing all 10 random samples from which a representative network was chosen. **A.** Top 100 buffering prediction networks from 10 samples of 100, 200 and 500 out of 1019 cell lines. Ensembl paralogs and non-paralogs are colored. **B.** Top 100 buffering prediction networks from 10 samples of 100, 200 and 500 out of 1019 cell lines. Low correlations in the original uncorrected data are colored.

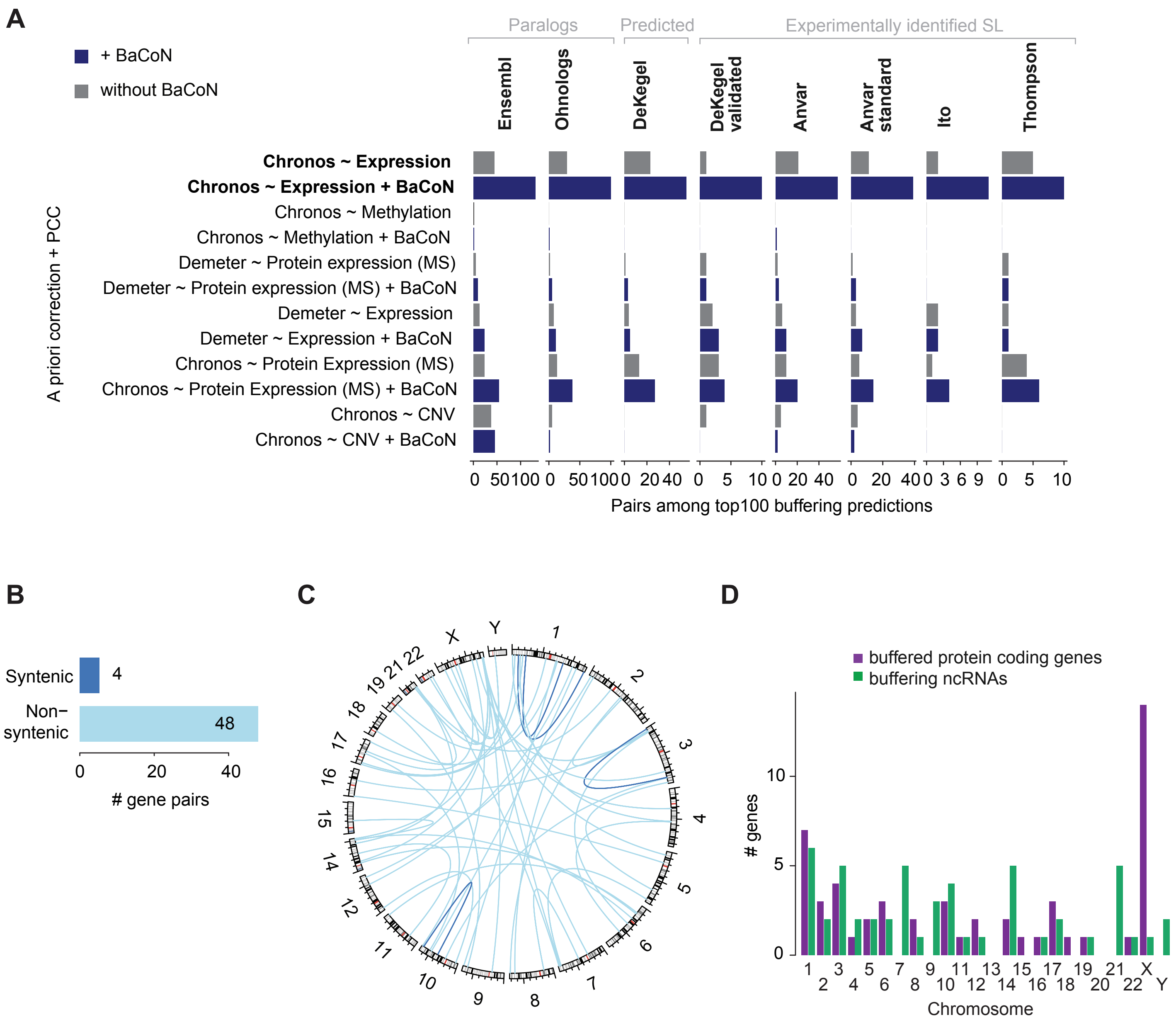

Appendix Figure S8. BaCoN performance for predicting gene buffering using different omics data sets from the Cancer Dependency Map. **A.** Performance of the different data**. B.** and **C.** Genomic location of paralog and non-paralog high-confidence buffering predictions. **D.** Number of buffered protein coding genes and buffering non-coding RNAs on each chromosome.
